# Supplementary material for: Natural Compound Boldine Lessens Myotonic Dystrophy Type 1 Phenotypes in DM1 Drosophila Models, Patient-Derived Cell Lines, and HSALR Mice
Source: Int J Mol Sci. 2023 Jun 6;24(12):9820. doi: 10.3390/ijms24129820 (PMC10298378; doi:10.3390/ijms24129820)
Supplement: Supplementary file 1 [file ijms-24-09820-s001.zip › ijms-2398427-supplementary.pdf]

Supplementary Materials

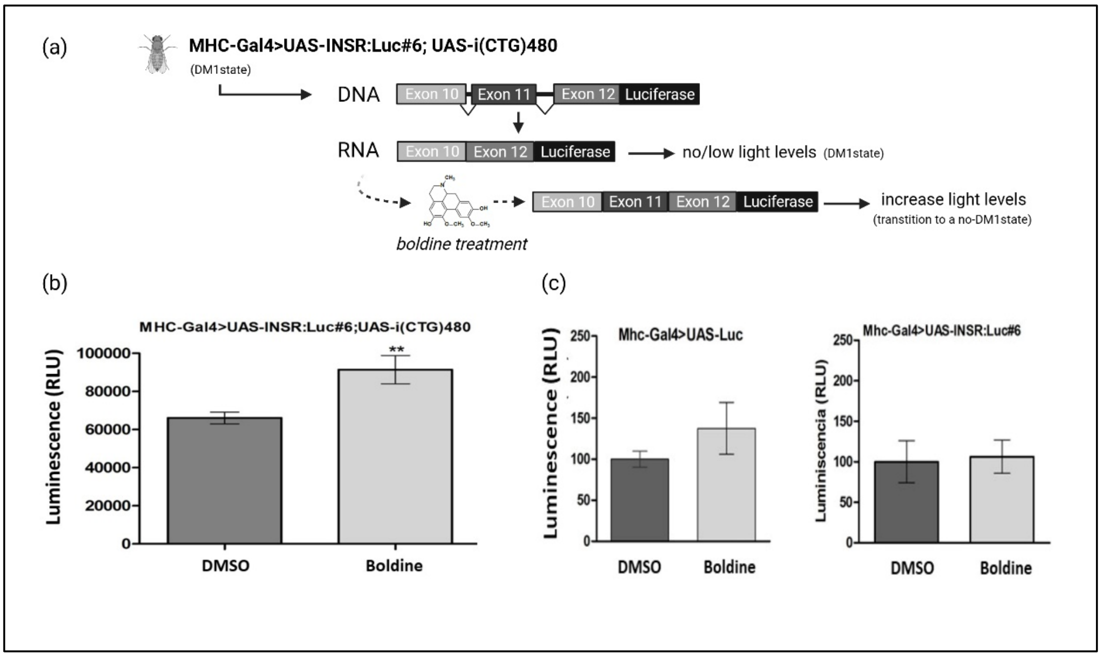

Figure S1. *DM1 spliceosensor system and validation of boldine activity in flies.* (a) Mhc-Gal4>UAS-INSR:Luc#6; UAS-i(CTG)480 DM1 spliceosensor flies increase levels of luciferase in response to small molecule treatments able to modulate AS of human INSR minigene to a no-DM1 state. (b)Treatment with boldine at 12.5 µM in spliceosensor flies that express CTG repeats showed a significant increase (**p-value<0.005) of the luciferase levels compared to control (flies treated with 0.25% DMSO). (c) Left: luminescence levels in Mhc-Gal4>UAS-Luc flies treated with boldine and DMSO. The value of statistical significance obtained (p-value <0.01) does not reach the levels established as different, which confirming that boldine does not interact non-specifically with luciferase. Right: luciferase activity in Mhc-Gal4>UAS-INSR:Luc#6 flies treated with boldine or DMSO. Not observed interference of boldine with the UAS/Gal4 expression system. In all cases the dose of boldine administered was the same used in the primary screening (12.5 µM). 0.25% DMSO was used as negative control. Measures calculated with the unpaired student's t test using the GraphPad program. Created with BioRender.com.

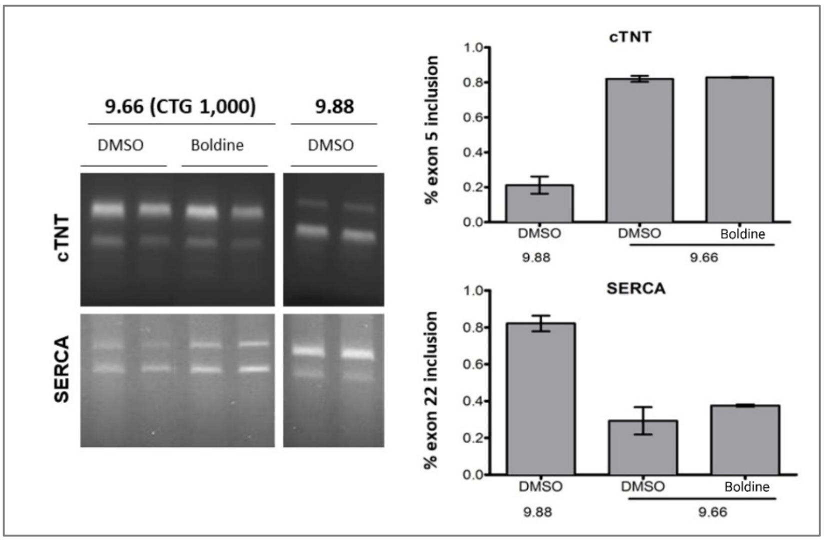

Figure S2. *Alternative splicing evaluation after boldine treatment in patient derived cells*. RT-PCR of the *cTNT* and *SERCA* genes from myoblasts of healthy individuals (9.88 line) or DM1 patients (9.66 line, carrier of 1,000 CTG repeats) treated with boldine (100 µM) or DMSO (1%). Treatment with boldine does not decrease the inclusion of exon 5 (top band) of the cTNT transcript as reflected in the % inclusion values in which the intensity of the lower band is quantified with respect to the upper one. Likewise, no significant increase in the % inclusion of *SERCA* exon 22 is observed. P-value calculated with the unpaired student's t test using the GrapPhad program. Created with BioRender.com.

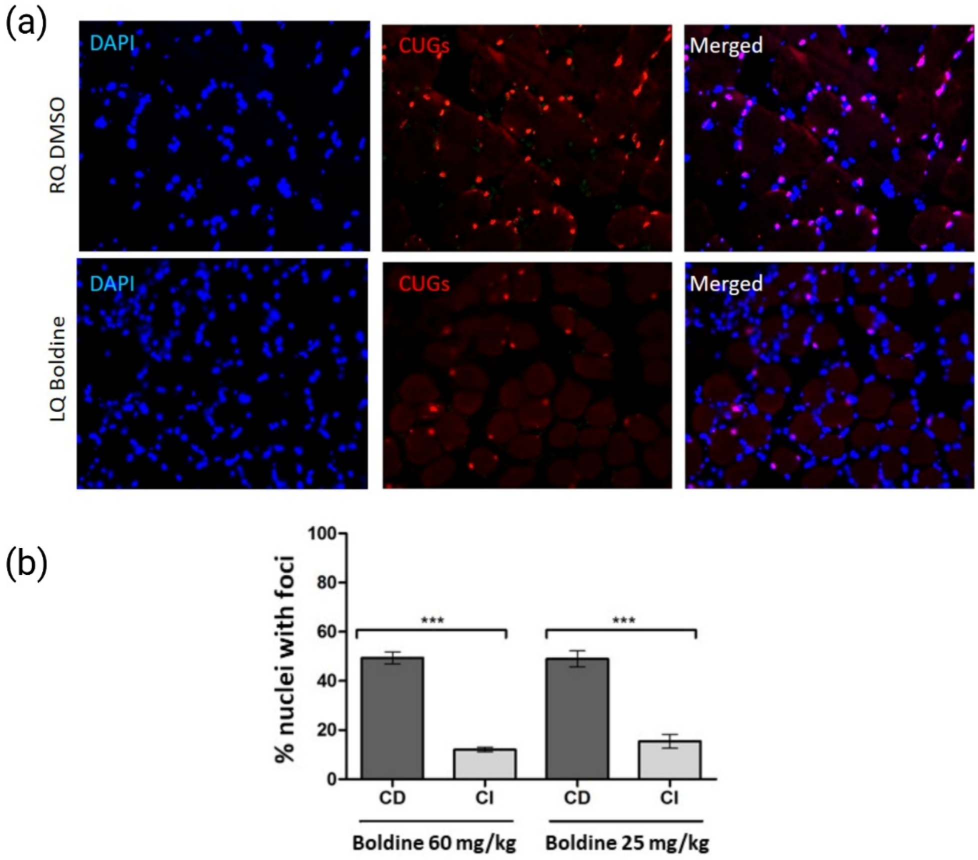

Figure S3. *Foci quantification in HSA^{LR} mouse mode*l. (a) Hybridization in fluorescent situ showing ribonuclear foci in cross sections of leg quadriceps hind legs of mice treated with boldine at 60 mg/kg in the left quadriceps (LQ, Boldine) and with DMSO (20%) in the right quadriceps (RQ, DMSO). Mice were treated daily with the compound or with DMSO for three consecutive days and sacrificed two days after the administration of the last dose. Nuclei are shown marked with DAPI in blue while foci are shown marked by the CAG probe in red. (b) Quantification of the number of nuclei with foci in sections of quadriceps after treatment with boldine at 60 mg/kg and 25 mg/kg or with DMSO. ***p-value<0.001. The quantifications of the number of nuclei with foci were carried out by counting at least 150 nuclei for each mouse (n=6 and n=3 for each condition in 60 mg/Kg and 25 mg/Kg, respectively). P-value calculated with the unpaired student's t-test using the GrapPhad program. Error bars correspond to the standard error. 63x optical microscope magnification. Created with BioRender.com.

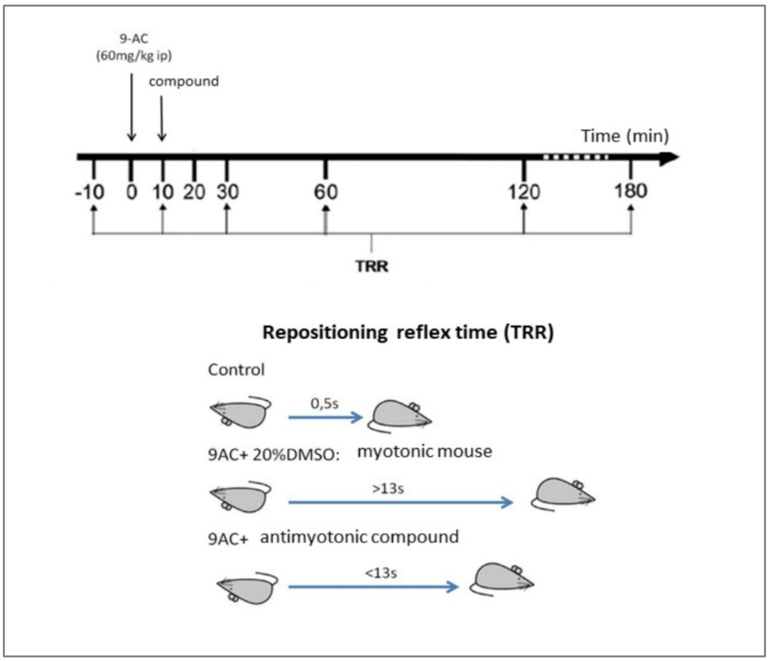

Figure S4. *Protocol for chemically inducible myotonia in mice and evaluation of candidate molecules*. The measurement of myotonia is performed by recording repositioning reflex time (TRR), which is the time it takes for a mouse to turn. Turned over on all fours after a supine position. The compound under study is administered 10 min after intraperitoneal injection of 9-AC. The RRT is measured 10 min before and 30, 60, 120, 180, and 240 min after injection of 9-AC. At each point, the TRR value is calculated as the mean of 10 measurements, leaving a minute of separation between each measurement. Under control conditions (before injection of 9-AC), the TRR is less than 0.5 seconds. After administration of 9-AC together with the vehicle (DMSO), the TRR increases to more than 13 seconds. According to this experimental design, an antimyotonic compound will reduce the TRR to less than 13 seconds [33]. Created with BioRender.com.

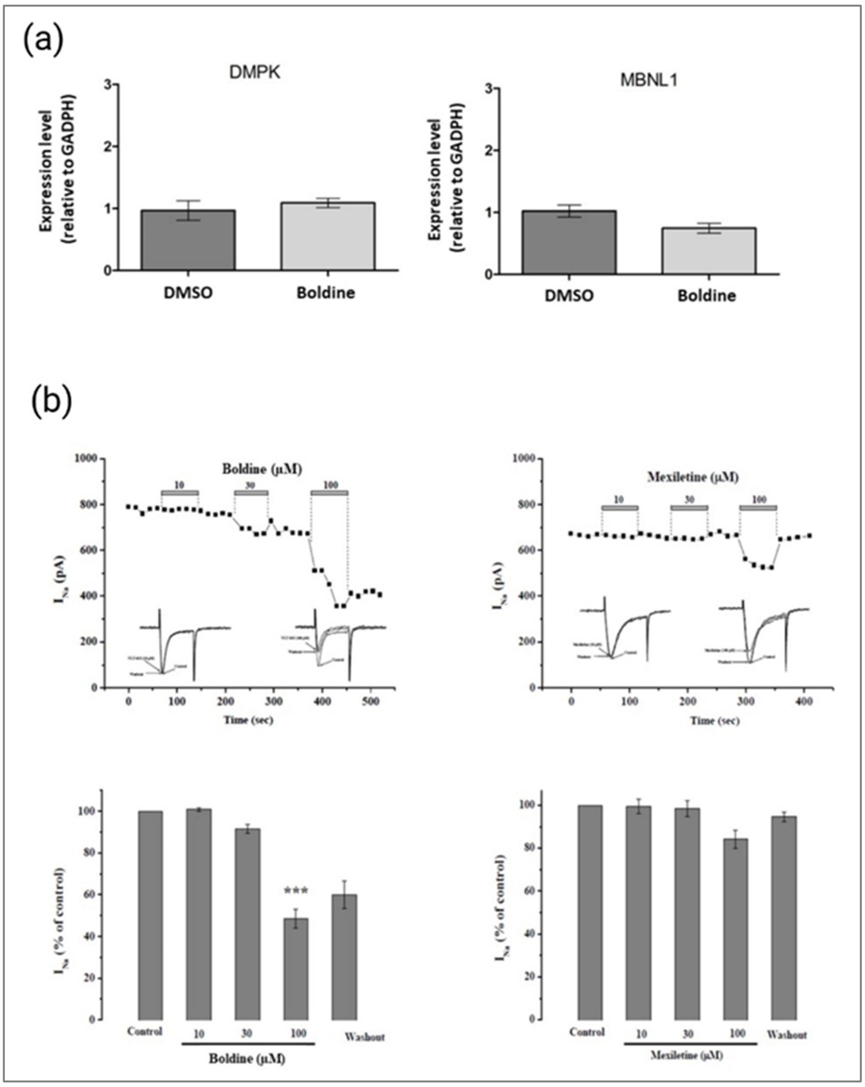

Figure S5. *Analysis of DMPK and MBNL1 gene expression levels and ion sodium membrane.* (a) qRT PCT of DMPK (A) and MBNL1 (B) in myoblasts from patients treated with DMSO (1%) (DMSO) or boldine a 100 μM (Boldine) normalized for GADPH expression. In both cases, no statistically significant differences between myoblasts treated with boldine or with DMSO. The error bars correspond to the standard error. (b) For the INa measurements, the membrane potential of the cells was set at -80 mV. The cells were applied test pulse at 0 mV (10 ms duration) every 15 s. The upper part shows the dose curves INa responses generated by the sequential 0 mv pulses. The application of boldine and mexiletine at 10, 30 and 100 μM is indicated by the upper horizontal bars. At the bottom is show standardized measures of INa blockade induced by boldine and mexiletine compared to the control without compound. Data from 8 cells from 3 different cultures were analyzed. ***p<0.005 regarding control. Test calculated with unpaired student's t test. Created with BioRender.com.

Table S1. Primers and amplification conditions for RT-PCRs

**RT-PCRs in mice samples**		
**Name**	**Sequence**	**Temp. and cycles**
Serca e21F	GCTCATGGTCCTCAAGATCTCAC	58ºC, 25 cycles
Serca e23R	GGGTCAGTGCCTCAGCTTTG	
Clc e6F	TTCACATCGCCAGCATCTGTGC	58ºC, 27 cycles
Clc e8R	CACGGAACACAAAGGCACTGAATGT	
Gadph F	ATCAACGGGAAGCCCATCAC	58ºC, 25 cycles
Gadph R	CTTCCACAATGCCAAAGTTGT	
**RT-PCRs in human cells**		
**Name**	**Sequence**	**Temp. and cycles**
SERCA R	GATGATCTTC AAGCTCCGGGC	58ºC, 25 cycles
SERCA F	CAGCTCTGCCTGA AGATGTG	
INSR F	CCAAAGA CAGACTCTCAGAT	55ºC, 35 cycles
INSR R	CAGCTCTGCCTGA AGATGTG	
cTNT F	GTCTCAGC CTCTGCTTCAGCATCC	55ºC, 29 cycles
cTNT R	ATAGAA GAGGTGGTGGAAGAGTAC	
GAPDH F	GTTCACACCCATGACGAACAT	55ºC, 27 cycles
GAPDH R	CATCTTCCAGGAGCGAGATC	
DMPK-F	GCTAGTGAAGATGAAGCAGACGG	
DMPK-R	GGAAGCACGACACCTCGC	
MBNL-F	TGCACAGAAATTAATGCGAACA	
MBNL-R	AATCATTTTCTCCTCGGTTGC	
